# MIB Guides: Measuring the Immunoreactivity of Radioimmunoconjugates

**DOI:** 10.1007/s11307-024-01898-x

**Published:** 2024-03-06

**Authors:** Samantha Delaney, Camilla Grimaldi, Jacob L. Houghton, Brian M. Zeglis

**Affiliations:** 1 Department of Chemistry, Hunter College of the City University of New York, New York, NY USA; 2 Program in Biochemistry, The Graduate Center of the City University of New York, New York, NY USA; 3 Department of Radiology, Memorial Sloan Kettering Cancer Center, New York, NY USA; 4 Department of Radiology, State University of New York at Stony Brook, 101 Nicolls Road, Health Sciences Center Level 4, Stony Brook, New York, NY 11794 USA; 5 Department of Radiology, Weill Cornell Medical College, 413 East 69th Street, New York, NY 10021 USA

**Keywords:** Antibody, Antibody fragment, Radioimmunoconjugate, Immunoreactivity, *In vitro* assay, Bead assay, Antigen binding

## Abstract

Immunoglobulins, both full-length antibodies and smaller antibody fragments, have long been regarded as effective platforms for diagnostic and therapeutic radiopharmaceuticals. The construction of radiolabeled immunoglobulins (*i.e.*, radioimmunoconjugates) requires the manipulation of the biomolecule through the attachment of a radiohalogen *or* the bioconjugation of a chelator that is subsequently used to coordinate a radiometal. Both synthetic approaches have historically relied upon the stochastic modification of amino acids within the immunoglobulin, a process which poses a risk to the structural and functional integrity of the biomolecule itself. Not surprisingly, radioimmunoconjugates with impaired antigen binding capacity will inevitably exhibit suboptimal *in vivo* performance. As a result, the biological characterization of any newly synthesized radioimmunoconjugate must include an assessment of whether it has retained its ability to bind its antigen. Herein, we provide straightforward and concise protocols for three assays that can be used to determine the immunoreactivity of a radioimmunoconjugate: (1) a cell-based linear extrapolation assay; (2) a cell-based antigen saturation assay; and (3) a resin- or bead-based assay. In addition, we will provide a critical analysis of the relative merits of each assay, an examination of the inherent limitations of immunoreactivity assays in general, and a discussion of other approaches that may be used to interrogate the biological behavior of radioimmunoconjugates.

**Supplementary Information:**

The online version contains supplementary material available at 10.1007/s11307-024-01898-x.

## Introduction

The remarkable specificity and affinity of antibodies and antibody fragments for their molecular targets have long made them enticing vectors for diagnostic and therapeutic radiopharmaceuticals. The construction of radioimmunoconjugates from immunoglobulins requires the modification of the biomolecule, either through the direct attachment of radiohalogens (*e.g.*, iodine-131 or astatine-211), the coupling of radiolabeled prosthetic groups (*e.g.*, the Bolton-Hunter reagent), or the bioconjugation of chelators (*e.g.*, desferrioxamine) that can stably coordinate radiometals (*e.g.*, zirconium-89). Historically, these cargoes have been attached to antibodies and antibody fragments via the stochastic modification of amino acids — most often lysines, cysteines, or tyrosines — that are distributed throughout the structure of the biomolecules. This approach has the potential to inadvertently alter an immunoglobulin’s complementarity determining regions and thus harm its ability to bind its antigen, an outcome that will inevitably impair the tumor-targeting capability of the final radioimmunoconjugate [1–4]. In recent years, many laboratories have sought to circumvent the inelegance of these stochastic methods by developing synthetic strategies that facilitate the attachment of chelators and radionuclides to specific sites within immunoglobulins. These site-specific and site-selective bioconjugation approaches not only offer better defined and homogeneous constructs but also drastically reduce the likelihood of attenuating the target binding capacity of immunoconjugates. Not surprisingly, site-specifically and site-selectively modified radioimmunoconjugates have been repeatedly shown to offer better *in vitro* and *in vivo* performance than their stochastically modified counterparts [5, 6]. Nonetheless, the conditions necessary for many of these more selective bioconjugation methods (*i.e.*, exposure to reducing agents or treatment with enzymes) still pose some risk to the integrity of fragile biomolecules [7].

A radioimmunoconjugate’s *in vivo* performance is inextricably linked to its ability to bind its antigen. Indeed, tumor-to-background image contrast and therapeutic indices can suffer even if only a portion of a population of radioimmunoconjugates exhibits reduced binding. It follows that an essential step in the fundamental characterization of any radioimmunoconjugate is the assessment of its ability to bind its target. This value is typically termed the “immunoreactivity” or “immunoreactive fraction” of the radioimmunoconjugate, with the former presented as a percentage (with a theoretical maximum of 100%) and the latter presented as a fractional value (with a theoretical maximum of 1.0). Before moving on, it is important to note that the term “immunoreactivity” is borrowed — imperfectly — from biology and immunology, where it is used to refer to the ability of a given antigen to provoke an immune response and to describe the ability of a substance to react with a given antibody [8]. The term has clearly come to have a different meaning in radiopharmaceutical chemistry, and a unique term would certainly decrease ambiguity and confusion during interdisciplinary collaborations, but upending the basic vocabulary of our field lies outside of the scope of this article. That said, we would support governing bodies such as the Society of Radiopharmaceutical Sciences in any efforts to update this terminology.

In this *Molecular Imaging and Biology Guide*, we will first provide detailed protocols (with representative sample data) for three assays that can be used to determine the immunoreactive fraction of a radioimmunoconjugate: (1) a cell-based linear extrapolation assay; (2) a cell-based antigen saturation assay; and (3) a resin- or bead-based assay. We will then discuss the relative advantages and disadvantages of each of these approaches as well as some of the limitations of immunoreactivity measurements as assays for the evaluation of radiolabeled immunoglobulins. Ultimately, our goal in writing this is to provide a single, critical compendium of these methods, thereby helping laboratories create standardized, robust, and reproducible protocols for the *in vitro* characterization of radioimmunoconjugates as they work to bring these valuable tools from the laboratory to the clinic.

## Procedures

### General Considerations

While the core component of each of the procedures described in this guide is a radiolabeled immunoglobulin, we will only provide protocols for the immunoreactivity assays themselves. Thankfully, there are several excellent extant reviews and protocols describing the synthesis and purification of radioimmunoconjugates [9–12]. It is recommended that each of these assays is performed at least in triplicate, as multiple replicates will help identify random experimental error and allow for the calculation of standard deviations. To provide representative data, we have performed each of the assays using a ^89^Zr-labeled variant of huA33 — [^89^Zr]Zr-DFO-huA33 — a humanized mAb that binds the transmembrane glycoprotein A33 that is expressed in > 95% of colorectal cancers. For the assays requiring cells, we have paired the radioimmunoconjugate with A33 antigen-expressing SW1222 human colorectal carcinoma cells acquired from American Type Culture Collection [13–15]. A list of required materials for each assay is available in the Supplemental Information.

### The Linear Extrapolation Assay (“The Lindmo Assay”)

**Fig. 1:**
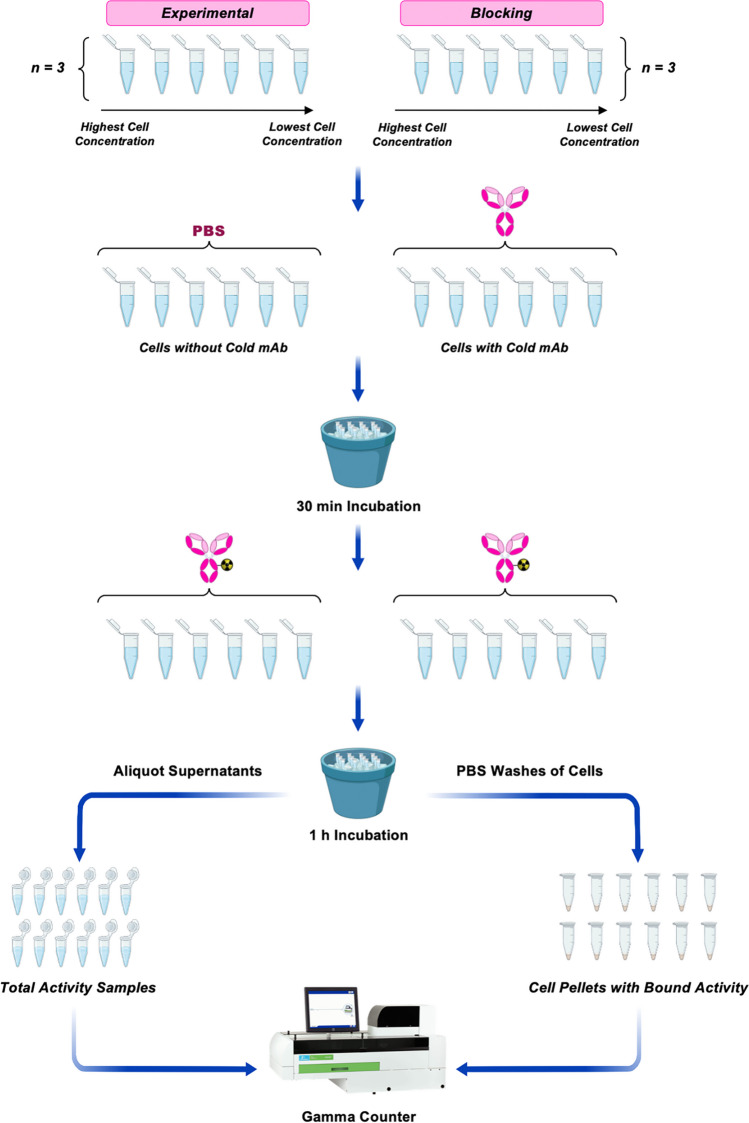
Schematic of the linear extrapolation assay

In 1984, Lindmo et al. first reported an assay for measuring immunoreactivity by determining the fraction of radioimmunoconjugate that binds to several concentrations of cells and then using a modified Lineweaver–Burk plot to extrapolate these results to a theoretical environment with infinite excess antigen (Fig. 1) [16]. A protocol is described below, our sample data is shown in Table 1, and a spreadsheet that can be used for this assay is available at 10.5061/dryad.mcvdnck6j. It is important to note that the cell numbers we describe below represent benchmarks and/or starting points but need not be the same in all experiments. Indeed, it may be necessary to adjust the number of cells based on the expression level of the target antigen (see “Discussion” for more).

1. In microcentrifuge tubes, prepare six aliquots of 5 × 10^6^ antigen-expressing cells in 1 mL PBS (pH 7.4) containing 1% BSA (PBS-BSA) (*n* = 3 for both the *experimental* and *blocking* cohorts).
2. Perform five 1:2 serial dilutions of the cell suspensions in PBS-BSA for each of the aliquots resulting in final volumes of 1 mL. The dilutions will contain 2.5 × 10^6^, 1.25 × 10^6^, 6.25 × 10^5^, 3.125 × 10^5^, and 1.5625 × 10^5^ cells.
3. Add 50 µg (3.33 × 10^−10^ mol) of non-radioactive immunoconjugate to the aliquots in the *blocking* series to saturate the antigens on the cells. Add the same volume of PBS to the *experimental* cohort to ensure that all the samples have the same volume.
4. Incubate all samples on ice for 30 min and manually agitate the tubes every 10 min via gentle inversion to prevent the formation of a cell pellet.
5. Prepare the radioimmunoconjugate to a final concentration of 40 ng/mL in PBS-BSA and add 500 µL to each sample.
6. At 1 h, remove a replicate sample (*e.g.*, 75 µL) from each aliquot in both the *experimental* and *blocking* series to serve as the total radioactivity aliquots.
7. Centrifuge the *experimental* and *blocking* samples at 650 rcf and remove the remaining liquid to isolate the cells.
8. Wash the cells 3 × with 1 mL PBS via centrifugation for 2.5 min at 650 rcf. Discard the supernatants.
9. Determine the counts-per-minute (CPM) of radioactivity in each sample using a gamma counter.

**Table 1:** Immunoreactive fraction values for [^89^Zr]Zr-DFO-huA33. Each experiment was performed in triplicate.

Assay	Immunoreactive fraction (mean ± SD)
Linear extrapolation assay	0.84 ± 1.0
Saturation assay	0.87 ± 1.9
Bead-based assay	0.85 ± 1.5

The count data collected via the protocol above should first be used to calculate the ratio of the cell-associated radioactivity to the total radioactivity for each sample. These ratios should then be plotted as a function of increasing cell concentration, and the plateau of the resulting curve denotes the immunoreactive fraction of the radioimmunoconjugate by approaching a value of 1. Alternatively — and more commonly — the same data can be graphed on a double-inverse plot. In this case, the ratio of the total radioactivity to the cell-associated radioactivity is plotted against the inverse of the cell concentration. Linear regression analysis can then be used to fit a straight line to these data. The inverse of the y-intercept represents the immunoreactive fraction (1/r).

In the event that the assay provides suboptimal immunoreactivity values — *i.e.*, values under 40–50% — it is (of course) possible that the antigen binding domains of the radioimmunoconjugate have been irreparably altered. However, this result could also be explained by a failure to include data from samples in which the radioimmunoconjugate is incubated with a sufficient excess of antigen. In this case, we recommend increasing the number of samples in the serial dilutions to provide more data points with high cell concentrations, a change that should result in a more distinct plateau and an improved linear extrapolation.

### The Saturation Assay

**Fig. 2:**
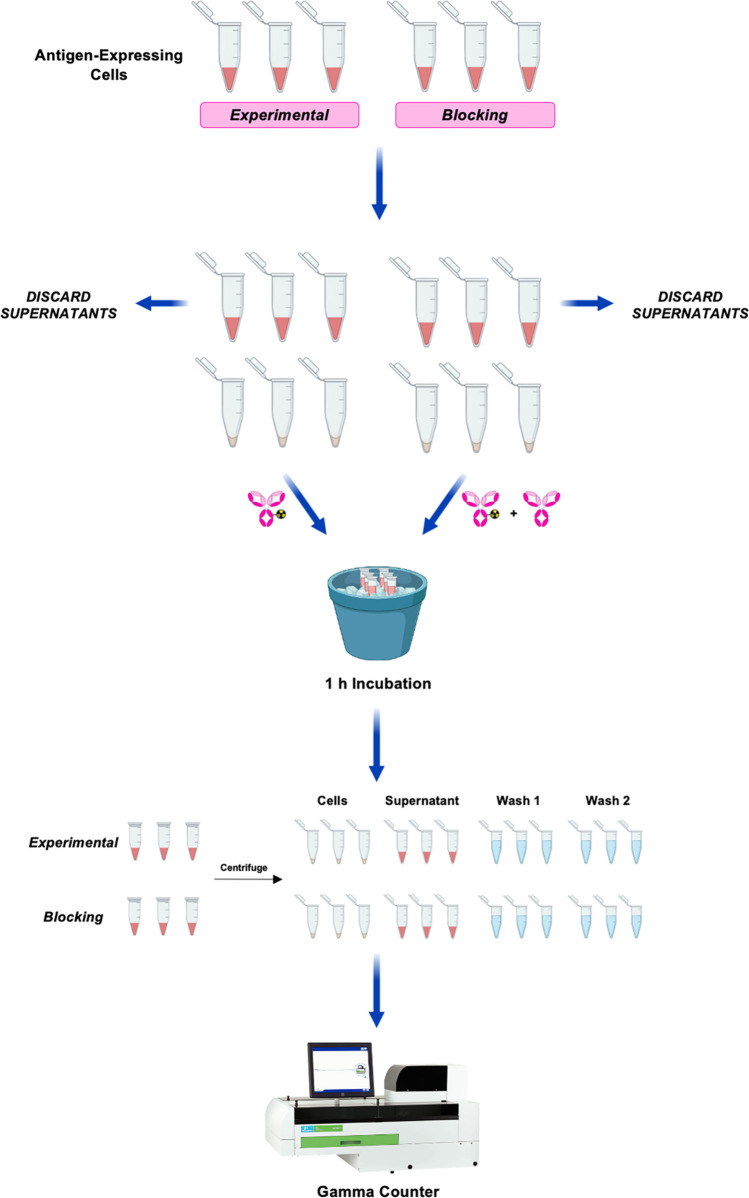
Schematic of the cell-based saturation assay

The cell-based saturation assay is the simpler, more straightforward cousin of the linear extrapolation assay described above. While the latter is predicated on the linear extrapolation of data to a theoretical condition of infinite antigen excess, the former relies upon single experimental samples that provide the radioimmunoconjugate in question with a vast excess of antigen (Fig. 2). This assay’s roots lie in a 1986 paper by Beaumier et al. in which the investigators sought to interrogate the binding of a radiolabeled monoclonal antibody to antigen-expressing melanoma cells [17]. A protocol is described below, our sample data is shown in Table 1, and a spreadsheet that can be used for this assay is available at 10.5061/dryad.mcvdnck6j. As we mention above, the cell numbers we describe represent benchmarks and/or starting points but need not be the same in all experiments. It may be necessary to adjust the number of cells based on the expression level of the target antigen (see “Discussion” for more).

1. Prepare six aliquots of 2 × 10^7^ cells (*n* = 3 for both the *experimental* and *blocking* cohorts) in microcentrifuge tubes.
2. Centrifuge the cells for 5 min at 650 rcf and remove the supernatants without disturbing the cell pellet.
3. Resuspend the cells in 200 µL of appropriate media.
4. Add 1 ng (6.67 × 10^−15^ mol) of the radiolabeled immunoconjugate to the *experimental* samples. Add 1 ng of the radioimmunoconjugate and 5 µg (3.33 × 10^−11^ mol) of unlabeled antibody to the *blocking* samples. The relative 5000-fold molar excess of non-radioactive antibody is necessary to saturate antigens on the cells.
5. Incubate the cells on ice for 1 h, manually agitating the tubes every 15 min via gentle inversion to prevent the formation of a cell pellet.
6. Prepare 18 additional microcentrifuge tubes: 6 labeled as *supernatant*, 6 labeled as *first wash*, and 6 labeled as *second wash*.
7. Following the incubation, centrifuge the cells for 2.5 min at 650 rcf.
8. Transfer the supernatants to the microcentrifuge tubes labeled as *supernatant*.
9. Add 500 µL of ice-cold media to the cell pellets, resuspend the cells, and re-centrifuge the cells for 2.5 min at 650 rcf.
10. Transfer the supernatants to the microcentrifuge tubes labeled as *first wash*.
11. Add 500 µL of ice-cold media to the cell pellets, resuspend the cells, and re-centrifuge the cells for 2.5 min at 650 rcf.
12. Transfer the supernatants to the microcentrifuge tubes labeled as *second wash*.
13. Determine the counts-per-minute (CPM) of radioactivity in each sample using a gamma counter.

The following equation may be used to determine the immunoreactive fraction (IF) of the radioimmunoconjugate:

$$\mathrm{I}\mathrm{F}=\frac{{\text{CPM}}_\text{Cells}}{{\text{CPM}}_\text{Cells}+{\text{CPM}}_\text{Supernatant}+{\text{CPM}}_{\mathrm{Wash}1}+{\text{CPM}}_{\mathrm{Wash}2}}$$

As with the linear extrapolation assay, the most obvious source of poor immunoreactive fraction values is a damaged radioimmunoconjugate. However, it is also possible that the abundance of the antigen on the cells in question is not sufficient to provide a dramatic excess with only 2 × 10^7^ cells. In this case, increasing the number of cells in the assay and/or decreasing the amount of radioimmunoconjugate employed may provide better results.

### The Bead-Based Assay

**Fig. 3:**
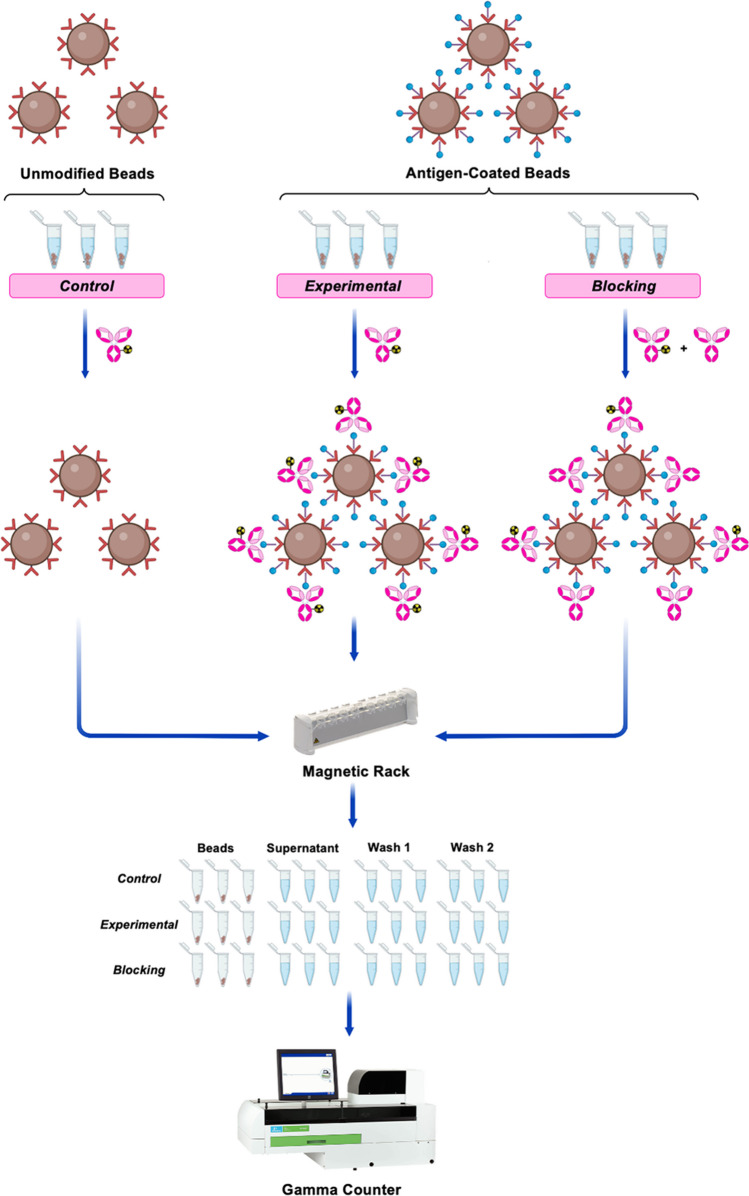
Schematic of the bead-based assay

In the first two assays, antigen-expressing cells are used as the means of interrogating the antigen-binding capability of the radioimmunoconjugate. While these assays are undoubtedly effective, cells are not always the most reliable, convenient, or cost-effective vehicle for the presentation of antigens. Indeed, even if we set aside the cost of cell culture and the persistent risk of cells dying, the expression of a given antigen by a cell line can vary significantly as a function of both passage number and growth conditions. Starting in the late 1990s and early 2000s, a handful of laboratories began reporting immunoreactivity assays in which the antigen in question was attached to a resin or bead rather than a cell [18–20]. More recently, Sharma et al. described a particularly well-designed assay predicated on attaching recombinant antigen bearing a His-tag to Ni–NTA-coated magnetic beads, though other adhesion methods —* e.g.*, biotin/streptavidin — could be used as well (Fig. 3) [21]. Ultimately, bead-based assays offer more control over antigen density than their cell-based cousins but, as we will discuss below, are limited to recombinant antigens that can be purchased or produced reliably. A protocol is described below, our sample data is shown in Table 1, and a spreadsheet that can be used for this assay is available at 10.5061/dryad.mcvdnck6j.

1. Prepare and label nine microcentrifuge tubes (*n* = 3 for the *control*, *experimental*, and *blocking* cohorts).
2. Add 20 µL of magnetic beads (12.5 mg/mL in 20% ethanol) to each of the tubes.
3. To wash the beads, add 380 µL of PBS (pH 7.4) with 0.05% Tween-20 and 50 mM Imidazole (PBS-T), vortex the tubes for 10 s, and quickly centrifuge the tubes to get any liquid off the underside of the lids.
4. Place the tubes on a magnetic rack for 30 s to allow the beads to move towards the rack.
5. While the tubes are still on the magnetic rack, remove and discard the supernatant from each of the tubes by pipetting.
6. Remove the tubes from the rack and repeat steps 3–5 with 400 µL of PBS-T.
7. Remove the tubes from the rack.
8. Add 390 µL of PBS-T to the *experimental* and *blocking* tubes. Add 400 µL of PBS-T to the *control* tubes.
9. Add 10 µL of a 0.1 mg/mL solution of antigen in PBS with 1% BSA to the *experimental* and *blocking* tubes.
10. Place all of the tubes on a rotating stand mixer and turn it up to the minimum speed that ensures that the solutions are mixed with each revolution.
11. Incubate the samples at room temperature for 30 min.
12. After incubation, place all the tubes on a magnetic rack for 30 s and remove and discard the supernatants.
13. Wash the beads by adding 400 µL of PBS-T, vortexing the tubes for 10 s, and then quickly centrifuging the tubes to get any liquid off the underside of the lids.
14. Place the tubes on a magnetic rack for 30 s to allow the beads to move towards the rack.
15. While the tubes are still on the magnetic rack, remove and discard the supernatant from each of the tubes.
16. Remove the tubes from the rack and repeat steps 12–14 with 400 µL of PBS-T.
17. Add 400 µL PBS-T and 1 ng (6.67 × 10^−15^ mol) of the radiolabeled antibody to the *control* and *experimental* tubes. Add 400 µL of PBS-T, 5 µg (3.33 × 10^−11^ mol) of unlabeled antibody and 1 ng of radiolabeled antibody to the *blocking* tubes.
18. Thoroughly vortex all tubes and place them on a rotating stand mixer at the minimum speed that ensures that the solutions are mixed with each revolution.
19. Incubate the samples while rotating at room temperature for 30 min.
20. Prepare 27 additional microcentrifuge tubes: 9 labeled as *supernatant*, 9 labeled as *first wash*, and 9 labeled as *second wash*.
21. After incubation, place all tubes on the magnetic rack for 30 s. Remove the supernatant from each tube, and pipette it into the appropriate *supernatant* tube.
22. Wash the beads 2 × with 400 µL PBS-T and pipet the supernatants from each of the washes in either the *first wash* tubes or the *second wash* tubes.
23. Determine the counts-per-minute (CPM) of radioactivity in each sample using a gamma counter.

The following equation may be used to determine the immunoreactive fraction (IF) of the radioimmunoconjugate:

$$\mathrm{I}\mathrm{F}=\frac{{\text{CPM}}_\text{Beads}}{{\text{CPM}}_\text{Beads}+{\text{CPM}}_\text{Supernatant}+{\text{CPM}}_{\mathrm{Wash}1}+{\text{CPM}}_{\mathrm{Wash}2}}$$

In a manner very similar to the cell saturation assay described above, one possible cause for low immunoreactivity values — beyond a damaged mAb — is the failure to present the radioimmunoconjugate with a vast excess of antigen. If this is the case, the situation may be alleviated by increasing the amount of antigen loaded onto the beads, increasing the number of beads used in the assay, and/or decreasing the amount of radioimmunoconjugate used in the assay.

## Discussion

Each of the assays described in this guide — the linear extrapolation assay, the cell saturation assay, and the bead-based assay — can be used to reliably and reproducibly determine the immunoreactive fraction of a radiolabeled immunoglobulin. Generally speaking, the immunoreactivity of a radioimmunoconjugate may be considered “acceptable” if the value exceeds 70%, though even lower thresholds have been used for clinical quality control measurements. Of course, much higher values (*i.e.*, 90% +) are the best-case scenario, but several factors — including the non-covalent nature of the immunoglobin-antigen interaction and the non-specific adhesion of radioimmunoconjugates and cells to tubes — practically mean that lower values are acceptable. If the immunoreactivity of a probe fails to meet these thresholds, it is, of course, possible that the antigen-binding domains of the immunoglobulin have been compromised during bioconjugation or radiolabeling. Along these lines, radiolytic damage to the immunoglobulin is a possibility. While radiolysis is unlikely in the context of radioimmunoconjugates labeled with positron-, gamma-, and low energy β-emitting nuclides, it may become an issue when working with isotopes with higher energy emissions, especially α-particles [22]. If radiolysis is suspected, the addition of a radioprotectant such as gentisic acid may ameliorate the problem. Disappointing immunoreactivity values may also mean that the assay itself needs optimization. For example, the immunoreactivity values produced by the bead-based assay can fall if there is insufficient loading of the antigen on the beads *or* if too few beads are used in the incubation itself. Likewise, cell-based immunoreactivity assays can yield very low values for antigens that are shed from living cells; in these cases, a bead-based assay can provide more reliable and straightforward data.

Each of the assays has its own set of strengths and weaknesses. Broadly speaking, these can be divided into logistical and scientific categories. Let us address the former first. The linear extrapolation assay is the most procedurally complicated and temporally demanding of the three, as it not only requires the use of cells but also a serial dilution of several samples to generate a single immunoreactivity value [23]. The cell saturation assay presents a slightly more streamlined option since it still requires cells but only a single tube per data point. Finally, it is tempting to surmise that the bead-based assay is the most straightforward approach, as it eschews cells and only requires a single tube per data point. In many cases, this is true. Furthermore, bead-based assays can be especially valuable in the context of antigens that are shed from living cells, as this phenomenon can complicate (or render moot) some cell-based assays [24]. But there is an important caveat here: bead-based assays are only possible if recombinant, tag-bearing versions of an antigen can be produced or purchased.

Perhaps not surprisingly, the scientific rigor of the three assays runs counter parallel to their procedural ease. The bead-based assay is predicated on an assumption that is often valid but occasionally dubious: that millions of beads present a small mass of radioimmunoconjugate with what is, effectively, infinite antigen. It is also possible that the recombinant antigens used for these assays could present epitopes that are inaccessible in their native, cell-expressed forms, meaning that a radioimmunoconjugate may bind to the former better than the latter (which would, of course, portend problems for its eventual *in vivo* performance). The cell saturation assay avoids concerns surrounding recombinant antigens but still relies on the same “infinite antigen” assumption; indeed, in this case, that assumption can be even more problematic, as cells offer even less control over the density of low abundance antigens than beads. Like its cell saturation counterpart, the linear extrapolation assay relies upon native antigens, but it eschews “infinite antigen” assumptions and instead relies upon the extrapolation of several data points to a theoretical “infinite antigen” scenario. Yet the linear extrapolation assay is not perfect. Along these lines, recent work by Denoel et al. provides a particularly cogent critical examination of the assays. Here, the investigators point out that the inclusion of datapoints for the lower cell concentrations could be a source of error in linear extrapolation of the double-inverse plot. To circumvent this issue, they recommend their Langmuir model-based “rectangular hyperbola” extrapolation method, arguing that this analysis will improve the rigor of the data [25]. Ultimately, given the aforementioned balance of strengths and weaknesses, we recommend using linear extrapolation assays for novel radioimmunoconjugates that have not previously been characterized, cell saturation assays for the quality control of previously characterized probes, and bead-based assays for the examination of radioimmunoconjugates that bind low abundance (*e.g.*, DLL3) or shed (*e.g.*, CA19-9) antigens.

Finally, it is important to note that immunoreactivity assays — like all assays — have limits and thus only represent one component within the proper characterization of a radioimmunoconjugate. In the context of a well-designed and optimized assay, a low immunoreactivity value is undeniably an ill omen for future *in vivo* performance. In contrast, a high value is encouraging, but does not necessarily guarantee optimal *in vivo* behavior. For example, Sharma et al. recently found that variants of ^89^Zr-labeled trastuzumab bearing increasing DOLs of desferrioxamine (*i.e.*, 1.4, 2.6, 4.0, and 10.9 DFO/mAb) boasted consistently high immunoreactivity values (> 80%). However, the radioimmunoconjugates with the highest DOLs demonstrated dramatically reduced tumor-to-liver activity concentration ratios compared to their more sparingly modified cousins. Surface plasmon resonance of the parent immunoconjugates unraveled the curious phenomenon: as the DOL increased, the binding affinities (*K*_D_ values), on-rates (*k*_a_), and “active concentrations” of the immunoconjugates all decreased [26]. In the context of this work, the immunoconjugate we employed in the assays described here — DFO-huA33 — did, in fact, boast a very similar *K*_D_ value to its unmodified parent mAb: 5.3 × 10^−9^ M and 3.0 × 10^−9^ M, respectively [27]. Yet the data by Sharma et al. clearly show that radioimmunoconjugates with “acceptable” immunoreactivity values can nonetheless have underlying problems. Consequently, we recommend complementing immunoreactivity assays with surface plasmon resonance analysis during the characterization of all novel radioimmunoconjugates.

## Conclusion

As radioimmunoconjugates continue to occupy a more prominent position within the nuclear medicine landscape, their thorough preclinical characterization and evaluation become all the more important. In the preceding pages, we have provided detailed protocols for a trio of methods for determining the immunoreactive fraction of these probes and described the advantages and disadvantages of each, with logistical ease and scientific rigor often offsetting one another. We have also addressed the broader limitations of immunoreactivity measurements and emphasized the importance of adding complementary analyses —* i.e.* by surface plasmon resonance — during the biological interrogation of new radioimmunoconjugates. Ultimately, it is our hope that this * Guide* serves as a critical source for information about these assays and, in so doing, helps streamline the preclinical development of our field’s next generation of effective diagnostic and therapeutic radioimmunoconjugates.

### Supplementary Information

Below is the link to the electronic supplementary material.

Supplementary file1 (PDF 150 KB)
