# Supplementary material for: MIB Guides: Measuring the Immunoreactivity of Radioimmunoconjugates
Source: Mol Imaging Biol. 2024 Mar 6;26(2):213–21. doi: 10.1007/s11307-024-01898-x (PMC10973015; doi:10.1007/s11307-024-01898-x)
Supplement: Supplementary file 1 — Supplementary file1 (PDF 150 KB) [file 11307_2024_1898_MOESM1_ESM.pdf]

Supplemental Information

MIB Guides: Measuring the Immunoreactivity of Radioimmunoconjugates

Samantha Delaney[1,2,3], Camilla Grimaldi[1,2,3], Jacob L. Houghton[4,*], Brian M. Zeglis[1,2,3,5,*]

[1]Department of Chemistry, Hunter College of the City University of New York, New York, NY, USA

[2]Ph.D. Program in Biochemistry, The Graduate Center of the City University of New York, New York, NY, USA

[3]Department of Radiology, Memorial Sloan Kettering Cancer Center, New York, NY, USA

[4]Department of Radiology, State University of New York at Stony Brook, Stony Brook, NY, USA

[5]Department of Radiology, Weill Cornell Medical College, New York, NY, USA

Corresponding Authors:

Jacob L. Houghton: 101 Nicolls Road, Health Sciences Center Level 4, Stony Brook, NY 11794; Phone: 631-444-5400; E-mail: jacob.houghton@stonybrookmedicine.edu

Brian M. Zeglis: 413 East 69th Street, New York, NY, 10021; Phone: 212-896-0433; E-mail: bz102@hunter.cuny.edu

Keywords: Antibody, antibody fragment, radioimmunoconjugate, immunoreactivity, *in vitro* assay, bead assay, antigen binding

METHODS

Synthesis of DFO-huA33

The huA33 antibody was diluted to a final concentration of 1.0 mg/mL in Chelex-treated PBS (pH 7.4). The pH of the solution was increased to 8.9 with 0.1 M Na_2CO_3. *p*-SCN-Bn-DFO in DMSO (10.0 mg/mL, 10 equiv.) was slowly added to the solution of antibody and thoroughly mixed. The mixture was incubated for 1 h on an agitating ThermoMixer at 37°C. After 1 h, the reaction was purified with size-exclusion chromatography (PD-10 Column; GE Healthcare; Chicago, IL, USA), and the final product was concentrated using a 2 mL Amicon Ultra centrifugal filter with a 50 kDa molecular weight cut-off (MilliporeSigma).

Radiolabeling

DFO-huA33 (0.2 mg) were diluted in Chelex-treated PBS (pH 7.4). $[^{89}Zr]Zr^{4+}$ [2.0 – 5.0 mCi] in 1.0 M oxalic acid was diluted with Chelex-treated PBS, and the pH of the solution was adjusted to 7.4 with 1.0 M Na_2CO_3. The mAb solution was combined with pH-adjusted zirconium-89, mixed thoroughly, and incubated on a ThermoMixer for 30 min at 37 °C and 500 rpm. The reaction was monitored using glass-fiber silica impregnated instant thin-layer chromatography (iTLC) paper (Pall Corp.; East Hills, NY, USA) using an EDTA solution as the eluent (50 mM, pH 5.5). The iTLC plates were analyzed on an AR-2000 radio-iTLC plate reader with WinScan Software (Bioscan, Inc.; Washington, DC, USA). Following the reaction, the $[^{89}Zr]$Zr-DFO-huA33 was purified using size-exclusion chromatography (PD-10 Column). Radiochemical purities were assayed using radio-iTLC with EDTA as the eluent (50 mM, pH 5.5).

Cell Culture

The human colorectal cancer cell line SW1222 was maintained under sterile conditions in Iscove's Modified Dulbecco's Medium supplemented with 10% heat-inactivated fetal calf serum, 2 mM L-glutamine, 100 units/mL penicillin, and 100 units/mL streptomycin. The cells were allowed to grow in a 37 °C environment with 5% CO_2 and were passaged every 7 days or upon reaching 80% confluency with 0.25% trypsin / 0.53 mM EDTA in Hank's Balanced Salt Solution without calcium and magnesium. All media was purchased from the Media Preparation Core at Memorial Sloan Kettering Cancer Center.

MATERIALS

General

All reagents were purchased from Fisher Scientific (Thermo Fisher Scientific, Waltham, MA, USA) unless otherwise noted. All phosphate buffered saline (PBS) solutions were diluted to 1× and filtered prior to use (Cat. No. BP3991). The huA33 antibody was produced by the Olivia Newton-John Cancer Research Institute as previously described [1-2]. The GPA33 antigen (Cat. No. 11277-H08H) was purchased from SinoBiological US Inc. (Wayne, PA, USA). Protein concentration measurements were performed via UV-Vis spectroscopy with an extinction coefficient of 2.1×10^5 M^{-1} cm^{-1} and a molecular weight of 1.5×10^5 Da. All water used was ultrapure (>18.2 $M\Omega cm^{-1}$ at 25 °C). *p*-SCN-Bn-DFO (Cat. No. B-705) was purchased from Macrocyclics, Inc. (Plano, TX, USA). ^{89}Zr was provided by 3D Imaging (Little Rock, AR, USA).

The Linear Extrapolation Assay

Reagents

- >3×10^7 cells of an antigen-expressing cell line
- >300 µg unmodified mAb
- 40 ng/mL radiolabeled mAb stock solution
- PBS
- BSA (CAT# BP1605-100)

Equipment

- Ice Bucket
- Eppendorf™ LoBind Microcentrifuge Tubes (Cat. No. 13-698-794)
- Eppendorf™ 5430R High-Speed Centrifuge
- PerkinElmer Wizard^2 γ-counter

The Saturation Assay

Reagents

- >1.2×10^8 cells of an antigen-expressing cell line
- >100 µg unmodified mAb
- >1 ng/mL radiolabeled mAb stock solution

- PBS
- BSA (Cat. No. BP1605-100)

Equipment

- Ice Bucket
- Eppendorf™ LoBind Microcentrifuge Tubes (CAT# 13-698-794)
- Eppendorf™ 5430R High-Speed Centrifuge
- PerkinElmer Wizard^2 γ-counter

The Bead-Based Assay

Reagents

- 0.1 mg/mL solution of antigen
- >100 µg unmodified mAb
- >100 ng/mL radiolabeled mAb
- PBS
- Tween 20
- Imidazole

Equipment

- HisPur™ Ni-NTA Magnetic Beads (Cat. No. 88831)
- Invitrogen™ DynaMagTM-2 Magnetic Rack (Cat. No. 12321D)
- Tube Revolver Rotator (Cat. No. 11-676-341)
- Eppendorf™ LoBind Microcentrifuge Tubes (Cat. No. 13-698-794)
- PerkinElmer Wizard^2 γ-counter

REFERENCES

1. King DJ, Antoniw P, Owens RJ, et al. (1995) Preparation and preclinical evaluation of humanised A33 immunoconjugates for radioimmunotherapy. Br J Cancer 72:1364-1372.
2. Lee FT, Hall C, Rigopoulos A, et al. (2001) Immuno-PET of human colon xenograft-bearing BALB/c nude mice using ^{124}I-CDR-grafted humanized A33 monoclonal antibody. J Nucl Med 42:764-769.
